# Relationship between emotion regulation strategies and total conviction in promoting behavior change

**DOI:** 10.3389/fpsyg.2022.941404

**Published:** 2022-08-24

**Authors:** Jun Shigematsu, Ryota Kobayashi

**Affiliations:** ^1^Department of Psychology, Faculty of Humanities, University of Toyama, Toyama, Japan; ^2^Department of Psychology, Graduate School of Education, Hiroshima University, Hiroshima, Japan; ^3^Department of Human Development and Education, School of Human and Social Sciences, Fukuoka Prefectural University, Fukuoka, Japan

**Keywords:** emotion regulation, cognitive reappraisal, total conviction, pain tolerance task, expressive suppression, distraction, behavioral change, distress tolerance

## Abstract

Research conducted in the recent past have proposed total conviction as a factor associated with cognitive reappraisal that may produce changes in emotion and behavior. However, the factors that influence total conviction are not yet clearly identified. In this study, we focused on daily emotion regulation strategies and examined the relationship between emotion regulation strategies and total conviction. A total of 42 undergraduate and graduate students participated in this study. They measured their tendency toward daily emotion regulation strategies and then engaged in the cold pressor task (CPT) which is a distress tolerance task. Participants were then presented with information that encouraged them to engage in the task while enduring distress, creating a context for cognitive reappraisal of the task. Thereafter, they engaged in a second CPT. Finally, the degree of total conviction to the information that prompted reappraisal was measured. The results showed that total conviction in the experimental situation predicted behavior change. We found that the tendency to use routine cognitive reappraisal was not associated with total conviction, while the tendency to use expressive suppression would have a negative effect on total conviction. Furthermore, the expressive suppression tendency was found to moderate the relationship between total conviction and behavior change. These results indicate that the occurrence of total conviction in cognitive reappraisal leads to behavior change, though the tendency toward daily cognitive reappraisal is not related to the occurrence of total conviction in the experimental setting. The results also suggest that daily expressive suppression inhibits total conviction, particularly in situations where cognitive reappraisal is required.

## Introduction

Gross (1998), in his study, proposed a process model of emotion regulation that assumes that emotions evolve over time, and those specific regulation strategies have various consequences at different stages of the emotion generation process. Furthermore, Gross and colleagues summarized strategies for controlling emotions (Gross and John, 2003; Gross, 2015). Typical strategies included cognitive reappraisal, expressive suppression, and distraction. Cognitive reappraisal is an antecedent-focused strategy, intended to alter the emotional impact of a situation by changing the way one thinks about it. For example, one might reevaluate a current difficulty as a challenge to personal growth rather than as a traumatic event. Expressive suppression is a strategy that focuses on emotional responses and attempts to suppress the behavioral expression of emotions. For instance, one might avoid expressing anger toward a friend or ignore feelings of anxiety. Thus, an individual might resort to distraction as a strategy to shift attention from negative feelings and thoughts to non-negative ones (Webb et al., 2012; Gross, 2013). For example, one might exercise or think about a forthcoming trip as a response to such negative feelings or thoughts.

Generally, cognitive reappraisal and distraction are known to be more effective at alleviating negative emotions and promoting mental health than expressive suppression (Aldao et al., 2010; Webb et al., 2012). It may particularly, have potential applications due to its positive effects on mental health. Principally, cognitive reappraisal and distraction are known to be more effective in alleviating negative emotions and promoting mental health than expressive suppression (Aldao et al., 2010; Webb et al., 2012). On the other hand, previous studies have found no significant differences in the effects of emotion regulation strategies (Zhu et al., 2019). Studies, like that of Soto et al. (2011), have shown that compared to individuals with Western cultural values, those with Eastern cultural values are more concerned about the potential inter-personal harm of inappropriate behaviors and tend to suppress their emotions, especially negative emotions, in various situations. Thus, although cognitive reappraisal strategies are by no means superior emotion regulation strategies in every context, the utility of cognitive reappraisal has, in fact, continued to attract attention in business (Côté, 2005), education (Davis and Levine, 2013), and health (Appleton et al., 2013).

Although the cognitive reappraisal strategy can positively impact mental health, attempting to implement this strategy does not always result in adaptive emotional and behavioral changes (Ford and Troy, 2019). In a previous study, nearly half of those who attempted reappraisal gave a response that corresponded to mid or lower level, when asked whether they had successfully reappraised (Ford et al., 2017). It has also been indicated that reappraisal does not always work positively, and that reappraisal is difficult for strong, emotionally arousing stimuli (Sheppes and Meiran, 2007; Sheppes, 2014).

One factor that makes cognitive reappraisal functional is total conviction, which is an experience-based cognitive manipulation that occurs when information that may be used to change behavior and emotion is available to an individual (Shigematsu et al., 2020). For example, a person might make a mistake at work and become depressed; then, thinking that they are incompetent, they might not challenge themselves at all. However, if the person’s manager reminded them that the knowledge obtained from this failure could be applied to the next job, this could promote the realization that one job failure does not mean a failure at all jobs. The employee’s depression might then alleviate, and they might begin to work more actively. Without total conviction, even if the individual knows theoretically that “this failure can be applied to the next job,” there might be no improvement in their mood or change in behavior. In this example, it is thought that total conviction occurs when information that enables the problem to be solved is presented; in this case, the information, “failure can be utilized in the next job,” can ensure a solution to the given problem. This is when mood improvement and behavioral change occur. In Shigematsu et al. (2020), total conviction was posited to not only manifest in behaviors such as those mentioned above but also in changes occurring in private events, such as during rumination. That is, when total conviction is not present, the individual is in a state of “I understand the theory, but it does not make sense to me,” and persistent iterative thinking about the problem occurs. One characteristic of total conviction is that it is often accompanied by an “aha” moment of realization and a physical sensation, such as feeling refreshed.

Ito (2009) studied the involvement of total conviction in cognitive reappraisal, focusing on the procedures of cognitive reappraisal. One of the typical induction procedures of cognitive reappraisal is to reevaluate the situation surrounding the stimulus or an aspect of the context (e.g., imagining that an image is fake or imagining that an apparently sick person in hospital will get better soon). Ito (2009) argued that when provided with information that may cause a reevaluation of a situation during an emotional arousal, both rational and logical understanding of the information and sincere conviction (i.e., total conviction) are conventionally included in the reevaluation strategy. Ito (2009) hypothesized that the difference between the former and the latter would affect the outcome of emotion regulation. Participants performed a cold pressor task, in which they had to endure pain by immersing their hands in cold water twice, post which, their pain tolerance time was measured. Between the first and second immersions, information that enduring pain has a positive effect on health was presented to encourage a reevaluation of the pain. After the second session, the participants were enquired if they were truly convinced of the statement. The results of this study showed that the time for which distress could be tolerated increased significantly from the first to the second session for the participants who were convinced of the truth of the statement. Thus, in contexts where cognitive reappraisal is required, total conviction is predictive of behavioral change.

However, research on the relationship between emotion regulation and total conviction is still in its early stages and thus, is very scarce. Although previous studies showed the occurrence of total conviction in situations requiring cognitive reappraisal promotes behavior change (Ito, 2009), they were limited as individual factors that promote total conviction during cognitive reappraisal have not been thoroughly examined. Therefore, the kind of individuals who will change their behavior with total conviction when they are placed in a situation that requires cognitive reappraisal are vaguely defined. Since it has been noted that there are individual differences in the effectiveness of cognitive reappraisal itself (e.g., Ford and Troy, 2019), examining individual factors of underlying total conviction may allow us to identify those for whom cognitive reappraisal is effective and those for whom it is not. This identification is essential as it plays a pivotal role in the practice of cognitive-behavioral therapy (Cognitive-behavioral therapy promotes cognitive restructuring, which refers to the transformation of maladaptive beliefs and automatic thoughts that maintain the problem; e.g., Beck et al., 1979). The process of cognitive restructuring involves a cognitive reappraisal because it is a strategy to intentionally produce a different way of thinking about a situation without fixing the way one thinks about it. It has been suggested that the patient’s total conviction is important in the treatment process, even during Cognitive Behavioral Therapy (CBT; Shigematsu et al., 2020). This study will contribute to the clinical practice of CBT by clarifying the factors that facilitate cognitive restructuring with total conviction.

In this study, we focused on the tendency to use daily emotion regulation strategies as individual factors related to the occurrence of total conviction. After measuring the participants’ daily emotion regulation strategies, we asked them to engage in a cold pressor task twice to measure their distress tolerance time as a behavioral measure (session 1 and session 2). Then, after session 1, information about the benefits of the cold pressor task was presented to the participants, thereby providing a context in which reevaluation of the cold pressor pain task could occur. Subsequently, we measured the total conviction regarding the presented information. Furthermore, we confirmed the relationship between total conviction and the change in distress tolerance time reported in previous studies. Cognitive reappraisal corresponds to the strategy in the cognitive change phase in Gross’s process model (Gross, 1998). Thus, it can be predicted that individuals who routinely use cognitive reappraisal are more likely to successfully achieve cognitive change through it. Since it is suggested that the process of achieving cognitive change involves the occurrence of total conviction (Shigematsu et al., 2020), we would expect individuals who routinely engage in cognitive reappraisal to also experience the occurrence of total conviction more often. This suggests that individuals who could utilize cognitive reappraisal regularly, may have a certain degree of cognitive flexibility and possess higher skills in understanding information experientially. Based on these arguments, it is predicted that even in the context in which cognitive reappraisal is encouraged in this study, those who routinely engage in cognitive reappraisal will be more likely to flexibly incorporate information that prompts reappraisal and generate total conviction. Contrarily, the routine use of distraction and expressive suppression is not expected to require the generation of total conviction for distraction and expressive suppression approaches. Thus, the propensity for distraction and expressive suppression was predicted to have no effect on total conviction. Examination of these hypotheses will lead to the identification of emotion regulation styles that influence the success of cognitive restructuring in CBT.

## Methods

### Participants

The final participants, excluding one dropout, were 42 native Japanese-speaking undergraduate and graduate students (11 males, 30 females, 1 other; M_*age*_ = 21.6 years, *SD* = 2.29). Participants were given a gift card worth 1,000 yen, worth approximately $8.7 (USD) for their participation. The study was reviewed and approved by the Research Ethics Committee of Hiroshima University. The participants provided written informed consent before their participation.^1^

### Materials

#### Task

We prepared a cold pressor task by referring to research conducted by Hayes et al. (1999) and Takahashi et al. (2002) to be undertaken by the participants. The task was to immerse a hand in cold water for as long as possible. Participants were instructed to put their hand in the cold water up to their wrist and keep it in the water as long as possible. The time spent in the water was defined as the pain tolerance time. The temperature of the cold water was 9°C.^2^ The task was performed twice (session 1 and session 2). The temperature of the room was maintained at 20–23°C.

For safety confirmation, blood pressure and pulse were measured before and after the session using a blood pressure monitor (dretec BM-100). Changes in mood were also confirmed by the participants verbally. In this study, no participants exhibited any change in blood pressure or pulse rate or deterioration in mood.

#### Instruction to promote reevaluation

A fake newspaper article explaining the usefulness of immersing hands in cold water was created (“Immersing hands in cold water is effective in preventing juvenile dementia”) as a material to encourage a reevaluation of the pain tolerance task. When the participants were asked immediately after the instruction whether they knew the contents of the newspaper article, all of them answered that this was the first time they had encountered the article. In this study, this instruction was used as information intended to induce reevaluation and thereby increase distress tolerance behavior.

#### Measures

##### Change in pain tolerance time

The increase in pain tolerance time from session 1 to session 2 of the cold pressor task was used as an index of behavioral change.

##### Comprehension of information

We asked the participants to rate the extent to which they understood the information in the fake newspaper article, using a four-point scale consisting of “not at all,” “not very much,” “mostly understood,” and “fully understood.”

##### Usefulness of the content of the information

After the participants read the article, we asked them to rate the extent to which they thought it was useful to dip their hands in cold water, using a 10-point scale ranging from “very much so” to “not at all so.”

##### Total conviction

To measure total conviction regarding the information about the usefulness of dipping hands in cold water, we asked, “Were you truly convinced by the information?” To make it easier for the participants to report their true feelings, we set aside time for casual talk and asked the question during this time. Specifically, after a post-experiment debriefing, tea was served to the participants, as they talked about how they felt during the experiment. This casual atmosphere reduced the risk of biases, such as social desirability bias, when answering the aforementioned question.

##### Tendency to use emotion regulation strategies

The Emotion Regulation Questionnaire (ERQ; Gross and John, 2003; Yoshizu et al., 2013) was used to assess cognitive reappraisal and emotion suppression tendencies. The ERQ-J comprised 10 items: a six-measure cognitive reappraisal (e.g., “I change my thoughts when I want to feel more positive”) and a four-measure emotional suppression (e.g., “When I feel positive emotions, I take care not so as to express them”). The responses were recorded with the help of a 7-point scale ranging from 1 (“Not at all applicable”) to 7 (“Extremely applicable”). Gross and John (2003) examined the reliability and construct validity of the ERQ and found high reliability and validity (α coefficient: reappraisal = 0.79, suppression = 0.73, test-retest reliability across 3 months was 0.69). The Japanese version of the ERQ (Yoshizu et al., 2013) reproduced the same factor structure as the original version, confirming its high reliability and validity (α coefficient: reappraisal = 0.77, suppression = 0.78, Test-retest reliability across 2 months was *r* = 0.61 for reappraisal and *r* = 0.65 for suppression).

The tendency to use distraction was measured using the positive refocusing subscale of the Cognitive Emotion Regulation Questionnaire (Garnefski et al., 2001; Sakakibara, 2015). Positive refocusing is defined as “thinking about pleasant and happy things instead of thinking about real events”; this scale fit the definition of distraction used in this study. Positive refocusing was assessed *via* four items (e.g., “I think about pleasant things that have nothing to do with that”). The response format was a 5-point scale ranging from 1 (“rarely”) to 5 (“always”).

### Procedure

The participants were asked to complete the emotional regulation strategy questionnaire immediately before the start of the experiment. During the main experiment, they were first asked to complete the initial cold pressor task (session 1). Thereafter, a fake newspaper article about the usefulness of immersing hand in cold water was shown to the participants. After that, they were asked to rate the usefulness of immersing their hands in cold water. The participants were then asked to perform the second cold pressor task (session 2). After session 2, a debriefing was conducted. The debriefing was followed by a casual talk, in which participants were asked to reflect on their state of mind at the time of instruction, and the degree of total conviction was measured regarding the newspaper information.

### Analysis

We calculated descriptive statistics, Cronbach’s alpha, Pearson’s correlation coefficients, single regression analysis, and multiple regression analysis using HAD 16.054 (Shimizu, 2016).

## Results

### Descriptive statistics and manipulation check

The descriptive statistics are shown in Table 1. The participants showed a high level of understanding of the instruction (mean = 3.69, standard deviation = 0.47) and indicated that they believed dipping their hands in cold water was useful (mean = 7.33, standard deviation = 1.73). Thus, we considered that the participants understood the content of the instructional text and that the information was conducive to increase in pain tolerance during the second pressor test.

**TABLE 1:** Descriptive statistics.

Variables	Mean	*SD*	Minimum	Maximum	Cronbach’s α
Cognitive reappraisal	28.74	4.95	18	39	0.717
Expressive suppression	14.53	4.84	4	23	0.779
Distraction	12.44	2.98	4	18	0.674
Pain tolerance time (session 1), [seconds]	82.91	47.29	18	242	–
Pain tolerance time (session 2), [seconds]	88.07	59.83	21	280	–
Increase in pain tolerance time, [seconds]	4.43	39.00	–60	160	–
Comprehension of information	3.69	0.47	3	4	–
Usefulness of the content of the information	7.33	1.73	4	10	–
Total conviction	3.67	1.03	2	5	–

### Simple regression analysis

A simple regression analysis was conducted to examine whether total conviction regarding information predicted a behavioral change. Specifically, the total conviction score for the instruction was used as the independent variable, and the increase in distress tolerance time between the first and second tasks was used as the dependent variable. The results showed that total conviction tended to predict the increase in pain tolerance (β = 0.299, *R*^2^ = 0.089, *p* = 0.055). This result indicates that total conviction predicted a behavioral change, as in previous studies.

### Multiple regression analysis

To test the hypothesis that the tendency to use emotional regulation strategies would affect total conviction, a multiple regression analysis was conducted with the three subfactors of emotional regulation strategies as independent variables and total conviction as the dependent variable. The results showed that the effects of the degree of “positive refocusing” and “reappraisal” on total conviction were not significant. Instead, the “suppression” strategy score had a negative relationship with total conviction (β = −0.443, *R*^2^ = 0.191, *p* = 0.005; Table 2).

**TABLE 2:** Multiple regression analysis of the relationship between emotion regulation strategies and total conviction.

Variables	Total conviction
Cognitive reappraisal	–0.014
Expressive suppression	−0.443**
Distraction	0.104
*R* ^2^	0.191*

**p < 0.01, *p < 0.05. The values are standardized regression coefficients.

**FIGURE 1:**
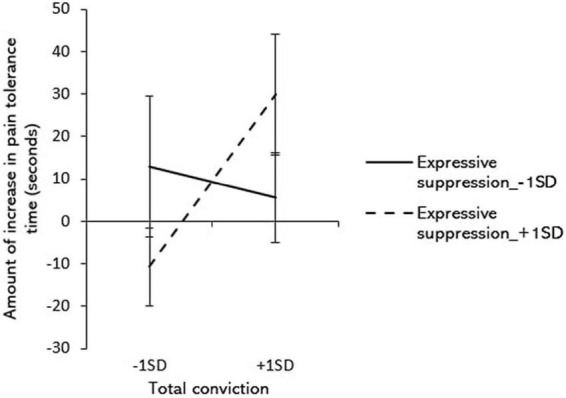
Moderating effects of inhibition on the influence of controlled and automatic cognitions on safety behaviors. Error bars are standard errors.

Since it can be assumed that daily emotion control strategies might modulate the relationship between total conviction and behavior change, interaction effect was additionally examined in an exploratory manner. Hierarchical multiple regression analyses were conducted to examine the moderating effect of expressive suppression on the relationship between total conviction about information and behavior change. The change in distress tolerance time was used as the objective variable, total conviction scores were regarded as explanatory variables in Step 1, and their interaction terms were entered in Step 2. In a simple slope test, the mean + 1 standard deviation of the moderating variable, expressive suppression, was considered high and the mean -1 standard deviation was considered low. The results showed significant interaction between total conviction and change in distress tolerance time (Table 3). Therefore, a simple slope test was performed (Figure 1), which indicated that when expressive suppression was high, the total conviction and distress tolerance time also increased (β = 0.52, *p* < 0.05). When expressive suppression was low, no significant relationship between higher total conviction and increased distress tolerance time was found (β = 0.48, *p* = 0.073). Furthermore, analyses with cognitive reappraisal and positive refocusing as moderating variables, respectively, showed no significant interaction between total conviction and change in distress tolerance time (reappraisal: β = 0.097, *p* = 0.54; positive refocusing: β = 0.041, *p* = 0.79).

**TABLE 3:** Hierarchical multiple regression analysis with expressive suppression as a moderator variable.

Dependent variable: Change in pain tolerance time			

	β	*R* ^2^	Δ*R*^2^
Step 1		0.089	0.089
Total conviction	0.301*		
Expressive suppression	0.006		
Step 2		0.164^+^	0.075
Total conviction	0.212		
Expressive suppression	0.004		
Total conviction × expressive suppression	0.287^+^		

*p < 0.05, ^+^p < 0.10.

## Discussion

The purpose of this study was to examine the relationship between individuals’ emotional regulation strategies and their total conviction in an experimental situation in which cognitive reappraisal was encouraged. The results did not support the hypothesis that the tendency toward everyday reappraisal strategies would predict total conviction in an experimental situation. One possible reason for the this is the discrepancy between the information and situation to be reevaluated in this experiment and the reevaluations that are likely to occur in everyday situations. For example, reevaluation of an experience of failure as “failure helps one’s growth” is often based on the experience of a task in which one committed oneself to failure. For example, Franz et al. (2021) provided an example of reevaluation when a student, upon receiving a “C” on a test, thinks, “This is just the first test of the semester, and there are plenty of opportunities to improve my grade.” There would be a greater ego-involvement for the target student than for the participants who received the information detailing the benefits of hand immersion in cold water as used in this study. In future studies, it will be necessary to reproduce and verify the same emotion regulation contexts as during daily reappraisals.

One notable result of this study is that the more likely participants were to use the expressive suppression strategy, the less likely they would experience total conviction when the context prompted cognitive reappraisal. All participants comprehended the usefulness of enduring the cold pressor task, but not to the point of total conviction. Individuals who were more likely to adopt inhibitory strategies may have had difficulty making positive sense of their distress; instead, total conviction likely promoted a positive evaluation. Several cross-sectional and laboratory-based studies have shown that expressive suppression is associated with increased oppositional deficits (Liverant et al., 2011; Zawadzki, 2015) and more severe symptoms of anxiety (Zawadzki, 2015). These results suggested that the individuals who were more likely to use expressive suppression daily, may have also accepted the information presented in the present experiment with excessive anxiety as well as suspicion, and continued to question the validity of the information. This phenomenon is the opposite of total conviction. Since there is some evidence for the effects of short-term emotion regulation through expressive suppression (Franz et al., 2021), it cannot be said that expressive suppression interferes with adaptive behavior. Still, the current results provide preliminary evidence that such suppression may prevent total conviction from occurring.

However, the results of hierarchical multiple regression analysis with expressive suppression as a moderating variable indicated that when expressive suppression was high, the total conviction and distress tolerance time also increased. This means that among those with a tendency toward expressive suppression, if the tendency is particularly high and if the context requires cognitive reappraisal, the behavioral change derived from the information is sufficiently significant to occur when total conviction is obtained. Based on Gross (1998), expressive suppression corresponds to the response modulation stage in the emotion regulation process. It is possible that those who tend to routinely use expressive suppression inhibition, were accustomed to suppressing reactions due to negative emotions, including distress. Such individuals succeeded in the cognitive reappraisal strategy with total conviction, especially, in the task of the present study. Moreover, they were also likely to succeed in the response regulation of “not reacting to pain,” and the interaction of emotion regulation in both the cognitive transformations; and the response regulation phases may have increased the distress tolerance time.

Thus, the results of the present study suggest that the tendency toward expressive suppression inhibits reappraisal with total conviction in response to the prompting of the experimental cognitive reappraisal strategy. Furthermore, those who have the skills to regulate their emotions and behavior to some extent, through expressive suppression, may change their behavior rather significantly through the interaction for reappraisal with total conviction.

In the present study, total conviction predicted behavior change as expected. This result highlighted the importance of total conviction in the cognitive operation of reappraisal and suggested the utility of total conviction in CBT to promote cognitive change (Shigematsu et al., 2020). However, since there is a dearth of empirical data, it is necessary to further examine the relationship between total conviction and cognitive change.

Furthermore, potential effects of Eastern and Western cultural differences may also explain the results of this study. The target population of this study was Japanese. People with Eastern cultural values tend to suppress negative emotions to avoid harming interpersonal relationships (e.g., Matsumoto et al., 2008). The habit of suppressing emotions for the sake of others may not have motivated emotional regulation when the context of experimental situation that demands reappraisal for one’s own sake about enduring distress, and it may have inhibited total conviction and behavior change in response to the information provided. So, differences in the effects of reappraisal and suppression of expression by culture may affect the ease of obtaining total conviction and behavior change in this study. Thus, further investigation is warranted to examine the same.

This study’s findings contribute to the field of emotion regulation research by providing suggestions to explain why and how emotion regulation strategies are selected and used. Gross (2015) updated the traditional process model by proposing an extended version. This is an attempt to update the model because the traditional process model does not explain how individuals select emotion regulation strategies. The present study shows that the tendency for expressive suppression is affected irrespective of whether total conviction is obtained during cognitive reappraisal or not. This implies that even if an individual is placed in a context in which cognitive reappraisal is beneficial, depending on his or her daily emotion regulation style, total conviction may not be obtained, and cognitive reappraisal may not be successful. This would be an experience of cognitive reappraisal failure and would affect the probability of choosing an emotion regulation strategy. Thus, by introducing the perspective of total conviction, the present study was able to suggest factors that influence the selection of emotion regulation strategies.

Furthermore, the clinical implication of this study is that it provided a basis for examining emotion regulation styles that influence the success of cognitive restructuring in CBT. Given the results of this study, individuals with inhibitory strategies may not always be easily successful with interventions that require cognitive reappraisal, such as cognitive restructuring methods, and thus may resort to acceptance and mindfulness-based intervention (e.g., Hayes et al., 2004) that may address inhibitory strategies.

## Limitations and future directions

There are a few limitations of this study. The sample size used in this study was inadequate. We used G*Power 3.1 (Faul et al., 2009) to check the sample size and to identify the appropriate level of sample size. A medium effect size (Effect size = 0.15) was set based on previous studies (Mizumoto and Takeuchi, 2008). Consequently, a sample size of about *N* = 77 was calculated to be desirable. In the future, it would be preferable to verify the validity of the sample size by referring to the results of the prior power analysis. It is unknown whether the participants used any strategies other than reappraisal, such as distraction, during the distress tolerance task; thus, the manipulation used in the current study was not optimal. Furthermore, as studies have compared the reappraisal and acceptance strategies (Troy et al., 2018), it would have been useful to include acceptance as an individual factor related to total conviction to expand the scope of the current findings. Another limitation is the low α coefficient of the distraction index. Future studies should thus, select more reliable indicators. Moreover, the index of total conviction used in this study needs to be validated to ensure that it comprehensively captures total conviction.

The present study represents a new foray into emotion regulation research that incorporates the concept of total conviction. In the future, it will be necessary to examine the mechanisms underlying the relationship between total conviction and emotion/behavior and the factors that promote total conviction after considering the aforementioned limitations of the current study. Furthermore, as the present study did not include a group without instruction, we cannot be sure whether the changes in the two cold pressor tasks were due to the instruction or other factors. Future studies with a control group will ensure the robustness of the findings of this study.

## Data availability statement

The raw data supporting the conclusions of this article will be made available by the authors, without undue reservation.

## Ethics statement

The studies involving human participants were reviewed and approved by Research Ethics Committee of Hiroshima University. The patients/participants provided their written informed consent to participate in this study.

## Author contributions

JS designed the study, collected and analyzed the data, and prepared the manuscript. RK reviewed and revised the manuscript. Both authors contributed to the article and approved the submitted version.

## Footnotes

1 The present study has some commonalities with “Development of the Checklist for Observing Client Responses to Cognitive Change in Cognitive Therapy-Focusing on ‘sense making”’ (Shigematsu, 2020), which was published in the Bulletin of the Graduate School of Humanities and Social Sciences, Hiroshima University, Studies in Education. However, the purpose, variables, and methods of analysis of the present study differ from those of the aforementioned paper.

2 Though water temperature in cold pressor tasks is often set within the range of 1–9°C, it is not consistent across studies. In this study, the water temperature was set at 9°C, which is the water temperature used in a previous study (Pena et al., 2017) and was stable due to the set room temperature and equipment availability.
